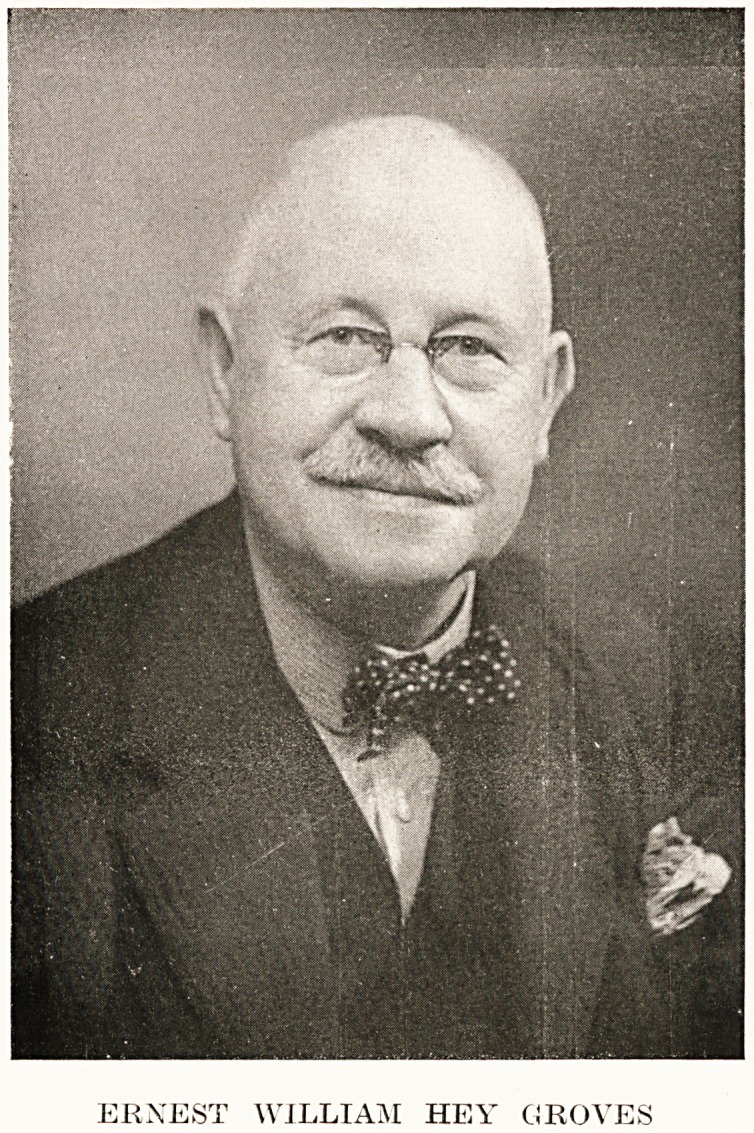# Ernest William Hey Groves

**Published:** 1945

**Authors:** 


ERNEST WILLIAM HEY GROVES

### Obituary

### ERNEST WILLIAM HEY GROVES, M.S., F.R.C.S.

ON 22nd October, 1944, Ernest Hey Groves died at his home in Clifton, Bristol, after a long and distressing illness, and there passed away with him a great English surgeon. He was born at Coonoor on the Nilgiri Hills in India on 20th June, 1872, the son of Edward Kennaway Groves, a Civil Engineer. When his father retired he settled in Bristol, bringing home with him his 3-year-old son. The boy had a will of his own, for it is related that on the steamer, finding his shoes too tight and pinching his feet he took them off and flung them overboard. He went to school at Redland Hill House, Bristol. Thence he gained an entrance scholarship at St. Bartholomew's Hospital, London, where he obtained the B.Sc. (London) in 1890, and qualified M.R.C.S., L.R.C.P., in 1895, taking the M.B. (London) in 1897 and the M.D. in 1900. He held the post of resident accoucheur at "Barts" under Sir Francis Champneys before entering upon general practice. He went first as assistant in various parts of the country and then settled into practice on his own account at Kingswood, Bristol. Here he performed many major surgical operations on private patients in his own house, where his wife assisted in the theatre and nursed the patients. Mrs. Hey Groves (*née* Frederica M. L. Anderson) had been a sister at "Barts" whom he married in 1896, and his success in establishing a reputation as an operating surgeon, whilst in general practice in the outskirts of Bristol, was in no small measure due to her competent help and encouragement. When, in 1903, a vacancy occurred on the surgical staff of the Bristol General Hospital, Hey Groves was invited to become Assistant Surgeon, an almost unprecedented recognition of the surgical attainments of a G.P. But by this time Groves had attained high academic honours, having taken the B.S. degree with honours in 1904, the M.S. with Gold Medal in 1905, and the F.R.C.S. in 1905—a remarkable record for a man engaged in busy practice. The judgement of the election committee of the Bristol General Hospital was amply justified by his previous record and still more so by his subsequent performance. Before long he had turned his attention to bone-surgery and became one of the pioneers in bone-grafting. His judgement was sound, his technique admirable. No one could be bolder when boldness was required, yet he could be as cautiously conservative as anyone upon occasions. In the War of 1914–18 he took a commission in the R.A.M.C. (T.F.) and went to Egypt in charge of the Surgical Division of a General Hospital during 1915–16. On returning to England he was attached as surgeon to the Beaufort War Hospital, Bristol. His friendship with and admiration for Sir Robert Jones were cemented by their common interest in orthopædic surgery. In was in orthopædic surgery that his reputation became world-wide. In 1935 he travelled to Australia for the B.M.A. Meeting, where he was President of the Orthopædic Section and was honoured by being made a Fellow of the Royal Australian College of Surgeons. In 1928–29 he was President of the British Orthopædic Association. At the Royal College of Surgeons of England he achieved notable recognition. He was Hunterian Professor in 1914, Jacksonian Prizeman in 1917, Arris and Gale Lecturer in 1917, Bradshaw Lecturer in 1926 and Hunterian Orator in 1930. He was a member of Council for twenty-three years and became a Vice-President of the College.

Perhaps one of his greatest triumphs was the making of the *British Journal of Surgery*. The germ of the idea in 1913 was his, though it was fostered from the beginning by the late Lord Moynihan. Here is an appreciation of his work as Editorial Secretary of the *Journal* from a former member of the publishing staff: "It was my good fortune to act as Secretary to Mr. Hey Groves for nearly twenty-five years in his work with the *British Journal of Surgery*. An Editor's task is not an enviable one, but it would have been hard to find anyone more energetic and understanding as the Editor of that *Journal* which he founded in 1913, and which owes its present high position in the surgical world very largely to his industry and far-sighted policy. His kindly criticism of articles sent in for publication, his helpful letters to young surgeons submitting perhaps for the first time, articles regarding the arrangement of which they were rather dubious, his advice about illustrative values, were all typical of his sincere resolve to maintain in surgical journalism the very highest standard. He was ever on the watch for articles dealing with ' everyday ' surgery, and long tedious tables of statistics did not greatly impress him. His search for " Critical Reviews " of such subjects as Tumours of the Lung, Surgery of the Sympathetic Nervous System, etc., brought in articles which have since been invaluable to research workers. He looked forward eagerly to the monthly meetings of the Editorial Committee which were held in the Examiners' Room of the Royal College of Surgeons (the very room in which the idea of the *B.J.S.* was born), his mind ever ready to answer any question ' arising out of the Minutes ' or to accept suggestions for the betterment of the *Journal*. The presentation to him in October, 1933, of a beaten silver salver (the work of Omar Ramsden) was a symbol of the gratitude and affection which the Editorial Committee and subscribers felt for him, and he prized both the gift and the spirit behind the gift, very highly. Lord Moynihan, in making the gift, said : ' This presentation is made to you on behalf of a number of subscribers to the *British Journal of Surgery* in recognition of your invaluable services during twenty years as Editorial Secretary. We offer you this salver and cheque as a tribute to your efficiency and devotion. You were one of those in whose mind was conceived the idea of founding in this country a journal worthy of the contributions to the Art and Science of Surgery which England and the British Empire were making. Difficulties were many, and opposition and indifference were found in high places. . . . In earliest days and throughout twenty years, with the exception of one year when you served in the Army overseas, our activities centred round you. All our plans, all our ambitions, all our efforts, found in you a most willing, most competent, and tireless worker. Your industry, your wise judgement, your enthusiasm and unwearying tact, have been of value beyond reckoning in maintaining year after year our corporate efforts. . . . To-day and by our simple gift we seek with our affection to assure you of our cordial recognition of your invaluable help.' "

In Bristol Groves was Professor of Surgery for ten years from 1922 to 1932, and his work was recognized by the University when the degree D.Sc. *honoria causa* was conferred upon him at the Centenary of the Bristol Medical School in 1933. Belfast, too, made him an Honorary LL.D. When he first became Professor he started clinical rounds in alternate weeks at the Hospital and the Infirmary, for he found the two institutions in something like opposition and rivalry, with the Infirmary students knowing nothing of the Hospital and vice versa. But beyond these public endeavours to promote harmony and co-operation, one who worked with him for eighteen years writes : " Possibly I know of some of his generous actions that few others are aware of. At least two successful medical men were enabled to complete their medical studies which otherwise they would, owing to financial difficulties, have had to abandon. He found out about their circumstances and paid the rest of their fees. Incidents he used to tell me about his own student days help to show the tenacity of purpose he had.

He won a scholarship for "Barts," and was sent to London to live with an uncle who resided at the far end of Hampstead. His uncle kept him, but did not allow him any pocket money. He had no money from home and so he used to get hold of an odd arm or leg from the Dissecting Room and smuggle it back to Hampstead, and in the basement by the light of candles, give grinds to younger students for a few shillings, but often he said there was a 'free' list for these grinds as well. Someone had to hold the door, for if they had been found out, they would have been sunk. Another incident was when a London surgeon asked him if he had ever seen London in the dawn, or before dawn broke, Mr. Groves said, 'I know the dawn in London as well as any surgeon you could name, I expect.' He said he was a devotee of Gladstone, and when he could he used to get into the House of Commons and listen all night to the Home Rule Bill, then walk back to Hampstead at 4 a.m., because, of course, there was no transport available at this time and he had no money for cabs. He said he did this many times. I cannot think of many modern students who would walk four to five miles in the chill of dawn to hear even Winston Churchill."

He was a prolific writer in the Journals and had published his well-known *Synopsis of Surgery*, also *Surgical Operations for Students and Nurses*, *Fractures*, and with Fortescue-Brickdale *A Textbook for Nurses.* His recreations were golf, swimming, motoring and travel. How he loved travel, and what a companion he was! Not the least of the attractions for him was the Moynihan Chirurgical Club. He called his travels "Busman's holiday," but it was not only to study surgery and surgeons in new places that he loved travelling. The novelty of other towns and other lands enthralled him. He was a dependable colleague, a cheery and delightful travelling companion and a grand friend. To his widow who survives him we offer our deep sympathy. *Requiescat in pace.*

## Figures and Tables

**Figure f1:**